# Evaluation of Longitudinal CD40 and CD40L Changes During Adjuvant Breast Cancer Therapy and Their Association with Left Ventricular Dysfunction

**DOI:** 10.3390/cancers18142340

**Published:** 2026-07-20

**Authors:** Georgia Efthymiou, Maria Anastasiou, Evangelos Oikonomou, Panagiotis Theofilis, Hector Katifelis, Elias Giallafos, Maria Gazouli, Anastasia Kotanidou, Gerasimos Siasos

**Affiliations:** 1 Laboratory of Biology, Department of Basic Medical Sciences, National and Kapodistrian University of Athens Medical School, 11527 Athens, Greece; gefthymiou@med.uoa.gr (G.E.); katifel@med.uoa.gr (H.K.); 2 Section of Medical Oncology, 2nd Propaedeutic Department of Internal Medicine and Clinical Research Institute, Attikon University Hospital, National and Kapodistrian University of Athens Medical School, 12461 Haidari, Greece; mariana@med.uoa.gr; 3 3rd Cardiology Department, “Sotiria” General Hospital of Chest Diseases of Athens, Athina Ioanni Martinou Endowed Chair, National and Kapodistrian University of Athens Medical School, 11527 Athens, Greece; boikono@med.uoa.gr; 4 1st Cardiology Department, “Hippokration” Athens General Hospital, National and Kapodistrian University of Athens Medical School, 11527 Athens, Greece; ptheofilis@med.uoa.gr; 5 3rd Department of Cardiology, Sotiria Chest Disease Hospital, National and Kapodistrian University of Athens Medical School, 11527 Athens, Greece; igialafos@med.uoa.gr (E.G.); gsiasos@med.uoa.gr (G.S.); 6 1st Department of Critical Care Medicine and Pulmonary Services, Evangelismos Hospital, National and Kapodistrian University of Athens Medical School, 10676 Athens, Greece; akotanid@med.uoa.gr

**Keywords:** anticancer therapy, anthracycline, trastuzumab, left ventricular dysfunction, CD40, inflammation

## Abstract

**Background**: Left ventricular (LV) dysfunction is a clinically important complication of anthracycline- and trastuzumab-based treatment in breast cancer. Inflammatory pathways may contribute to cancer therapy-related cardiac dysfunction, and the CD40/CD40 ligand (CD40L) axis has been implicated in vascular inflammation and myocardial injury. This study assessed longitudinal changes in CD40 and CD40L during adjuvant treatment and their association with LV dysfunction. **Methods**: Twenty-eight women with operable breast cancer receiving adjuvant anthracycline-based chemotherapy with or without trastuzumab were prospectively evaluated. Blood samples were collected at baseline, 6 months, and treatment completion at 15 months to measure CD40 and CD40L. Cardiac function was assessed by transthoracic echocardiography, including left ventricular ejection fraction and global longitudinal strain, at baseline and every 3 months. Repeated-measures analysis of variance examined temporal biomarker changes and interactions with LV dysfunction. **Results**: Participants had a mean age of 52.6 ± 10.7 years and a mean BMI of 25.8 ± 4.8 kg/m^2^; 64% were postmenopausal, 76% had HER2-positive disease, and 79.3% received radiotherapy and trastuzumab. During follow-up, 15 patients (53.6%) developed LV dysfunction. CD40 levels remained stable overall (*p* = 0.31), whereas CD40L increased significantly over time (*p* < 0.001). Baseline CD40 did not differ by LV dysfunction status (*p* = 0.79). However, CD40 showed a significant time-by-LV dysfunction interaction (*p* = 0.022), increasing late in patients with LV dysfunction and declining in those without it. CD40L showed no such interaction (*p* = 0.85). **Conclusions**: In this small exploratory cohort, CD40 demonstrated differential longitudinal patterns according to subsequent LV dysfunction, whereas CD40L increased over time without a clear association with LV dysfunction. These findings should be interpreted as hypothesis-generating and require validation in larger prospective cohorts.

## Simple Summary

Some breast cancer treatments can affect the heart, even when they are effective against cancer. Detecting early signs of heart injury during treatment is important so that doctors can monitor patients more closely and reduce the risk of long-term heart problems. This study examines whether changes in two blood markers linked to inflammation, CD40 and CD40 ligand, are associated with the development of reduced heart function in women receiving treatment for breast cancer. The authors aim to determine whether repeated measurement of these markers during therapy can help identify patients who may be at higher risk of treatment-related heart dysfunction. If confirmed in larger studies, these findings may support the development of better monitoring strategies in cardio-oncology and improve understanding of how inflammation contributes to heart injury during cancer treatment.

## 1. Introduction

The application of anthracyclines, taxanes, and HER2-targeted therapies such as trastuzumab in the adjuvant treatment of breast cancer has had a significant impact, reducing rates of recurrence and mortality. However, these developments have also led to increased cardiovascular complications, significantly worsening the prognosis of breast cancer survivors [1,2,3]. Therefore, identifying patients at higher risk for heart disease is crucial in cardio-oncology. However, numerous cardiac biomarkers have not reliably demonstrated the ability to foresee these risks [3,4,5,6].

Among the mechanisms implicated in anthracycline- and/or trastuzumab-induced myocardial injury, oxidative stress, mitochondrial dysfunction, apoptosis, and chronic inflammation appear to play important roles [6].

A signaling pathway that has attracted significant attention is the CD40/CD40 ligand (CD40L) pathway [7]. CD40 is present on several cell types, including endothelial cells, monocytes, platelets, and cardiomyocytes; conversely, CD40L is primarily found in activated T lymphocytes and platelets [8]. Activation of this pathway promotes the production of inflammatory cytokines, endothelial dysfunction, oxidative stress, and thrombosis [8]. Beyond systemic inflammation, the CD40/CD40L signaling pathway has also been implicated in endothelial activation, oxidative stress, myocardial fibrosis, and adverse cardiac remodeling. Experimental studies suggest that CD40-mediated immune activation may contribute to myocardial injury through activation of NF-kB signaling, macrophage recruitment, and amplification of inflammatory cascades within cardiac tissue. These mechanisms may be particularly relevant in cancer therapy-related cardiotoxicity, where chronic inflammatory and vascular injury pathways appear to contribute to progressive myocardial dysfunction [7].

Elevated circulating levels of soluble CD40L have been associated with adverse cardiovascular outcomes, atherosclerosis, and heart failure [9,10]. Additionally, experimental findings indicate that CD40 signaling contributes to myocardial remodeling and inflammatory cardiac injury [11]. However, there is limited information regarding the behavior of the CD40/CD40L axis in breast cancer patients receiving potentially cardiotoxic therapies.

Although established biomarkers such as cardiac troponins and natriuretic peptides are currently used in cardio-oncology surveillance, inflammatory biomarkers reflecting immune-mediated myocardial injury remain insufficiently explored. Therefore, investigation of the CD40/CD40L axis may provide additional mechanistic and prognostic insights regarding cancer therapy-related cardiac dysfunction [12,13].

In this study, we investigate the variations in circulating CD40 and CD40L levels in patients with operable breast cancer undergoing anthracycline-based chemotherapy, either with or without trastuzumab, and how these variations correlate with the occurrence of left ventricular dysfunction during treatment.

## 2. Materials and Methods

### 2.1. Patient and Blood Samples

A total of 28 patients with resectable breast cancer were evaluated after breast surgery (conservative or mastectomy) and before adjuvant chemotherapy initiation, which consisted of 4 cycles of epirubicin 75 mg/m^2^ and cyclophosphamide 600 mg/m^2^ every 14 days and thereafter 12 weekly cycles of paclitaxel 80 mg/m^2^. Following chemotherapy completion, intravenously administer trastuzumab at a dose of 6 mg/kg every 3 weeks, continuing for one year in total.

Medical history and blood samples were collected at three predefined time points: (1) before initiation of adjuvant chemotherapy (baseline, time point A), (2) 6 months after treatment initiation (time point B), and (3) at the end of the predefined study follow-up (15 months after treatment initiation, time point C).

At the baseline visit (time point A) and every 3 months, echocardiographic evaluation was performed until the end of trastuzumab therapy (15 months after chemotherapy initiation, time point C).

All patients enrolled in the study (1) had breast cancer histologically confirmed; (2) they were treated with anthracyclines and with or without trastuzumab as adjuvant chemotherapy, and (3) had normal left ventricular systolic performance at baseline with ejection fraction (LVEF) > 50%. Patients with (1) systematic inflammatory disease or infection; (2) hepatic or renal failure; (3) coronary artery disease; (4) other cardiovascular disease; and (5) uncontrolled arterial hypertension were excluded. Subjects were enrolled from the Department of Clinical Therapeutics, Alexandra Hospital, National and Kapodistrian University of Athens Medical School between May 2015 and July 2019.

Ethical approval for the study was obtained from the Ethics Committee of “Hippokration” Athens General Hospital (Approval Code: 8028; Approval Date: 17 June 2015). All participants provided written informed consent prior to enrollment, including specific authorization for the collection and use of blood samples and related data for research purposes.

### 2.2. Biochemical Analysis

Blood samples were collected in serum separator tubes, and before centrifuging at 3000 rpm for 10 min at 4 °C, were left to clot for 30 min. Serum was aliquoted and stored at −80 °C without repeated freeze–thaw cycles. All analyses were performed using the same reagent batches. Soluble CD40 and its ligand (CD40L) were measured by enzyme-linked immunosorbent assay (ELISA) using the Human Tumor necrosis factor receptor superfamily member 5 (CD40) ELISA Kit (Assay Genie Ltd., Dublin, Ireland) and the Human CD40L (Cluster of Differentiation 40 Ligand) ELISA Kit (FineTest Biotech Inc., Boulder, CO, USA), respectively, according to the manufacturer’s instructions. High-sensitivity cardiac troponin I and N-terminal pro B-type natriuretic peptide (NTproBNP) were measured using commercially available assays as part of the clinical evaluation.

### 2.3. Assessment of Left Ventricular Function

The same experienced operator performed all echocardiographic examinations in a dimly lit room. A Vivid E cardiovascular ultrasound system (GE Healthcare, Milwaukee, Wisconsin, USA) equipped with a 2.0–3.6 MHz harmonic phased-array transducer was used. Two-dimensional and Doppler echocardiographic measurements were obtained according to the recommendations of the American Society of Echocardiography (ASE)/European Association of Cardiovascular Imaging (EACVI). Left ventricular (LV) dimensions were assessed from the parasternal long-axis view [14]. Left ventricular ejection fraction (LVEF) was calculated using the modified Simpson’s biplane method [14].

Global longitudinal strain (GLS) was assessed offline using two-dimensional speckle-tracking echocardiography with dedicated EchoPAC software v204 (GE Healthcare, Horten, Norway). GLS analysis was performed from standard apical two-, three-, and four-chamber views, and values were calculated according to current ASE/EACVI recommendations [14].

A reduction in LVEF of 10 percentage points to an LVEF of 40–49% and/or a relative decline in GLS of 5% was defined as LV dysfunction, corresponding to the occurrence of symptomatic or asymptomatic impairment in LV systolic function during follow-up.

### 2.4. Statistical Analysis

All statistical calculations were performed in SPSS software (version 27.0; SPSS Inc., Chicago, IL, USA). Continuous variables were tested for normality via the Kolmogorov–Smirnov test and through visual inspection of P-P plots. Variables not normally distributed underwent logarithmic transformation to improve normality. Accordingly, they are presented as mean ± standard deviation or median (interquartile range) for normally and non-normally distributed data, respectively. Categorical variables are presented as percentages. Differences between categorical variables were tested by forming contingency tables and performing chi-square tests. For continuous variables, the *t*-test was used for between-group differences and repeated measures analysis of variance for changes over the follow-up period. Differences were considered statistically significant when the *p*-value was less than 0.05.

## 3. Results

### 3.1. Baseline Characteristics of the Study Population

The study population consisted of 28 women with breast cancer, with a mean age of 52.6 ± 10.7 years. The mean body mass index (BMI) was 25.8 ± 4.8 kg/m^2^. Overall, 64% of patients were postmenopausal, and 36% were premenopausal (Table 1).

Regarding cardiovascular risk factors, hypertension was present in 32% of patients, dyslipidemia in 28%, and diabetes mellitus in 4%. A family history of CAD was reported in 17.2%, and a family history of malignancy in 13.8%. Concerning smoking status, 20% were current smokers, 16% former smokers, and 64% had never smoked.

With respect to tumor characteristics, most patients (76%) had HER2-positive disease, while 24% were HER2-negative. Disease stage was II in 60% and III in 40% of patients. Radiotherapy was delivered to 79.3% of patients. Targeted therapy with trastuzumab was administered in 79.3%.

### 3.2. Oncologic Treatment and Changes in CD40-CD40L

**Figure 1:**
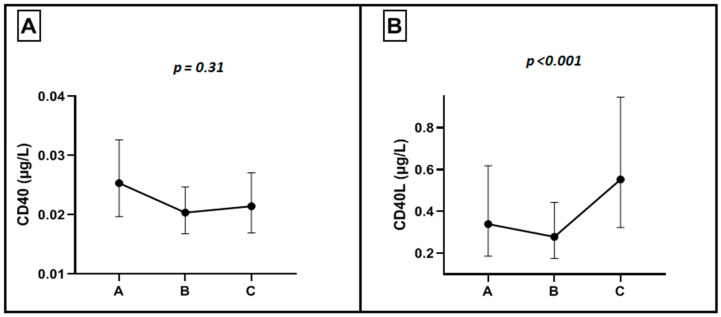
Changes in (**A**) CD40 and (**B**) CD40L during follow-up in patients undergoing treatment for breast cancer. Time points A, B, and C represent baseline (time point A), 6 months after treatment initiation (Time point B), and the end of trastuzumab therapy (15 months after treatment initiation) (Time point C). The reported *p*-values represent the overall effect of time derived from repeated-measures analysis of variance across the three study time points. Data are presented as geometric mean with 95% CI.

In the overall study population, circulating CD40 levels remained stable throughout follow-up, with no significant temporal variation observed in the study population [0.021 (0.016–0.039) μg/L at baseline (time point A), 0.021 (0.016–0.027) μg/L at the second time point (B), and 0.020 (0.015–0.027) μg/L at the final time point (C); *p* = 0.31] (Figure 1A).

In contrast, CD40L levels demonstrated significant changes over time, increasing from 0.219 (0.089–1.777) μg/L at baseline (time point A) to 0.234 (0.105–0.711) μg/L at the second time point (B) and 0.536 (0.141–2.650) μg/L at the final time point (C). Repeated-measures analysis confirmed a statistically significant temporal trend in circulating CD40L concentrations (*p* < 0.001) (Figure 1B).

### 3.3. Occurrence of LV Dysfunction During Oncologic Treatment

During follow-up, 15 patients (53.6%) developed LV dysfunction. No statistically significant differences were observed in baseline clinical and demographic characteristics between patients who developed LV dysfunction and those who did not. No significant differences were observed regarding family history of cancer or coronary artery disease, menopausal status, or cardiovascular risk factors, including hypertension, diabetes mellitus, and dyslipidemia (all *p* > 0.05) (Table 2).

Importantly, most cases of LV dysfunction corresponded to subclinical CTRCD detected through serial echocardiographic surveillance and GLS alterations, while symptomatic heart failure was not observed during follow-up.

### 3.4. LV Dysfunction and CD40/CD40L

**Figure 2:**
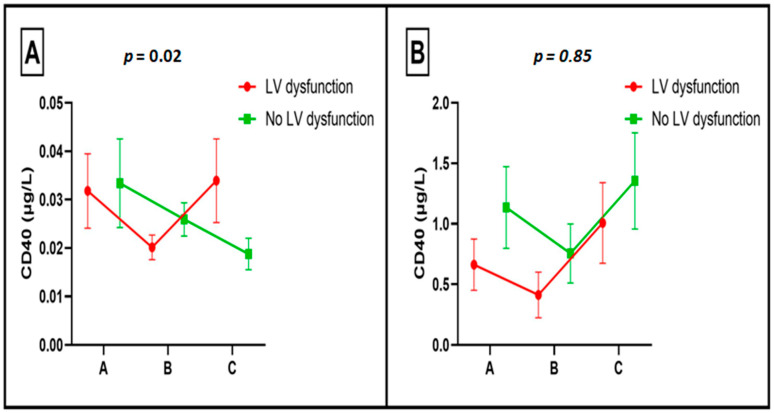
Changes in (**A**) CD40 and (**B**) CD40L and their association with the occurrence of LV dysfunction during follow-up in patients undergoing treatment for breast cancer. Time points A, B, and C represent baseline (time point A), 6 months after treatment initiation (Time point B), and the end of trastuzumab therapy (15 months after treatment initiation) (Time point C). The reported *p*-values represent the time × LV dysfunction interaction obtained from repeated-measures analysis of variance, assessing whether the longitudinal biomarker trajectories differed between patients with and without LV dysfunction. Data are presented as geometric mean with 95% CI.

Baseline CD40 levels did not differ significantly between patients who developed LV dysfunction and those who did not (0.021 (0.015, 0.040) μg/L vs. 0.023 (0.018, 0.034), respectively, *p* = 0.79). However, a statistically significant interaction between time and the occurrence of LV dysfunction was observed (*p* = 0.022), indicating a differential temporal pattern of CD40 levels between the two groups. Specifically, in patients without LV dysfunction, CD40 levels showed a gradual decrease over time, whereas in those with LV dysfunction, an increase was observed at the final point (Figure 2A). In contrast, no significant interaction between time and LV dysfunction was found for CD40L levels (*p* = 0.85), suggesting a similar temporal pattern of change in both groups (Figure 2B).

We also exploratorily assessed the potential contribution of trastuzumab exposure to the association between longitudinal CD40 and CD40L trajectories and the development of LV dysfunction. The three-way interaction between CD40, LV dysfunction, and trastuzumab exposure was not statistically significant (*p* = 0.36), with similar findings for CD40L (*p* = 0.32).

## 4. Discussion

In the present study, longitudinal changes in circulating CD40 and CD40L levels in breast cancer patients receiving anthracycline-based therapy were studied, and their association with the development of LV dysfunction was explored. The principal findings of the present study are that LV dysfunction develops in a substantial proportion of breast cancer patients receiving anthracycline- and trastuzumab-based therapy, while circulating CD40L levels increase significantly during treatment. Moreover, patients who developed LV dysfunction exhibited a gradual increase in circulating CD40 levels, suggesting a potential association between the CD40/CD40L axis and treatment-related cardiac impairment.

The development of LV dysfunction during cancer treatment has important prognostic implications. Cancer therapy-related cardiac dysfunction is associated with an increased risk of symptomatic heart failure, interruption or discontinuation of potentially life-saving oncologic therapy, reduced quality of life, and increased long-term cardiovascular morbidity and mortality [13,14]. Identification of patients at higher risk for LV dysfunction at an early stage is essential in cardio-oncology [15], as closer surveillance may allow cardioprotective interventions and may lead to improved cardiac recovery and continuation of optimal anticancer treatment [3]. Current surveillance strategies in cardio-oncology primarily rely on imaging modalities and conventional cardiac biomarkers such as troponins and natriuretic peptides. However, these approaches may not fully capture immune-mediated and inflammatory mechanisms contributing to myocardial injury. In this context, inflammatory biomarkers associated with endothelial dysfunction and immune activation may provide complementary information regarding susceptibility to CTRCD [12,13].

Cardiotoxicity continues to pose limitations in contemporary breast cancer therapy, and this is also relevant in patients treated with anthracyclines and HER2-targeted therapies [16]. Assessment of LV systolic performance based on echocardiography, and specifically on LV ejection fraction and LV deformation through GLS assessment, represents the cornerstone of surveillance [3]. However, these imaging modalities often detect myocardial impairment only after functional alterations have already occurred [3], while other advanced imaging modalities are not always feasible in all clinical settings and may also lack sensitivity for the early detection of cardiotoxicity [17]. Therefore, circulating biomarkers with the potential to identify subclinical myocardial injury or predisposition towards myocardial impairment attract research and clinical interest [12].

Beyond a net clinical impact based on their potential diagnostic and prognostic utility, studying circulating biomarkers also provides important mechanistic insights into the biological pathways involved in cancer therapy-related cardiotoxicity. Additionally, mechanistic studies improve our understanding of the molecular and inflammatory processes that drive myocardial injury and adverse cardiac remodeling. Inflammatory mediators, including interleukin-6 (IL-6), tumor necrosis factor-α (TNF-α), and C-reactive protein (CRP), have been implicated in cancer therapy-related cardiac dysfunction and have been associated, but not specifically, with myocardial injury, adverse ventricular remodeling, and heart failure progression [18,19]. The CD40-CD40L signaling axis is a well-established mediator of immune activation, and is associated with endothelial dysfunction, oxidative stress, and vascular inflammation, biological processes and pathways that may contribute to myocardial remodeling and progression toward heart failure phenotype [7,8,20].

In our study, circulating CD40L levels increase over follow-up. Activated platelets and immune cells may drive the release of CD40L, reflecting systemic inflammatory activation and linking to adverse cardiovascular outcomes [8,21]. Higher levels of CD40L were observed at the end of the anticancer treatment, suggesting the cumulative inflammatory and immune activation of prolonged exposure to anticancer therapy. Nevertheless, CD40L kinetics were not associated with the development of LV dysfunction, possibly expressing systemic inflammatory response rather than myocardial dysfunction per se. The observed increase in circulating CD40L levels may additionally reflect platelet activation and vascular inflammatory responses induced by anthracycline- and trastuzumab-related endothelial injury. Experimental evidence suggests that CD40L-mediated signaling contributes to oxidative stress amplification and endothelial dysfunction, processes closely linked to adverse myocardial remodeling [20].

Interestingly, levels of CD40 were associated with LV dysfunction. CD40 levels declined during follow-up in patients who did not show evidence of LV dysfunction. In the opposite direction, CD40 levels were increased in the group of patients identified with LV impairment. This opposite pattern may be clinically meaningful, as sustained inflammatory activation could represent an early pathophysiological signal of adverse myocardial remodeling and reduced cardiac adaptation to cardiotoxic treatment. Persistent CD40 activation may promote myocardial inflammation through activation of NF-kB-dependent signaling pathways, enhanced cytokine production, and recruitment of inflammatory immune cells into cardiac tissue. Such mechanisms could contribute to impaired myocardial adaptation during prolonged exposure to cardiotoxic therapies [11]. However, the present study has no mechanistic analyses to confirm activation of these pathways. Therefore, the proposed biological mechanisms remain speculative.

Interestingly, CD40 levels at baseline are not associated with subsequent LV dysfunction. This finding suggests that changes over follow-up in CD40 levels may offer greater prognostic value than a time point or baseline measurement. In this direction, biomarker assessment at multiple time points could identify patients with evolving myocardial injury even before overt clinical deterioration. Expectantly, such an approach may support more individualized surveillance and earlier identification of patients at higher risk.

Nevertheless, the present findings should be interpreted in the context of certain limitations. This was a single-center observational study with a relatively small sample size, limiting statistical power and generalizability, and rendering our findings as preliminary and exploratory. In addition, the limited sample size precluded multivariable analyses adjusting for potential confounders. Accordingly, even nonsignificant interaction tests should not be interpreted as evidence of the absence of effect. Furthermore, established cardiac biomarkers, including troponins and natriuretic peptides, were not assessed longitudinally at the same time points as the biomarkers investigated in this study, precluding direct comparative analyses. Therefore, the incremental prognostic or diagnostic value of CD40 beyond established biomarker-based surveillance is beyond the scope of this study. In addition, mechanistic validation at the tissue or molecular level was not available; therefore, the biological pathways linking CD40 signaling with myocardial dysfunction remain speculative. Furthermore, other inflammatory biomarkers, such as Interleukin -6 [18], tumor necrosis factor alpha (TNF-α), and C-reactive protein were not assessed to provide a more comprehensive characterization of inflammatory potential, combined evaluation with the CD40/CD40L axis. Importantly, cases of LV dysfunction corresponded to subclinical CTRCD identified through serial echocardiographic and GLS monitoring rather than overtly symptomatic heart failure. Finally, longer follow-up (to determine reversibility or not of the cardiac dysfunction) and external validation in larger prospective cohorts are required to determine the prognostic and clinical utility of CD40 in cardio-oncology surveillance strategies.

In terms of clinical relevance, the potential added value of CD40 should be viewed as mechanistic rather than immediately clinical. Echocardiographic surveillance using LVEF and GLS identifies functional myocardial impairment, while troponins and natriuretic peptides primarily reflect myocardial injury and hemodynamic stress. In contrast, the CD40/CD40L axis may provide information on immune activation, endothelial dysfunction, platelet activation, oxidative stress, and inflammatory remodeling. Therefore, CD40 may be most useful as part of a multimarker strategy rather than as a standalone screening tool. However, at present, the available evidence is insufficient to support the routine clinical use of CD40 for monitoring cardiotoxicity. Nevertheless, the observed longitudinal changes in CD40 may provide mechanistic insights and may guide further investigation of this pathway. The present study supports further evaluation of this pathway. Future studies with larger prospective cohorts are required to validate the prognostic role of CD40 in cardio-oncology, in an attempt to individualize surveillance strategies and improve early identification of patients at higher risk for cancer therapy-related cardiac dysfunction.

## 5. Conclusions

In breast cancer patients treated with anthracycline- and trastuzumab-based regimens, we observed longitudinal changes in biomarkers associated with the CD40/CD40L signaling pathway during therapy. Although circulating CD40L levels varied significantly over time in the overall cohort, CD40 was the only marker that showed a distinct longitudinal pattern in relation to the development of LV dysfunction. Patients who developed LV dysfunction demonstrated a progressive rise in circulating CD40 levels, whereas those with no evidence of LV dysfunction showed a gradual decline during follow-up. These results are hypothesis-generating and support further investigation of the CD40/CD40L axis in larger prospective studies incorporating troponins, natriuretic peptides, standardized CTRCD phenotyping, and adequately powered analyses by treatment exposure.

## Figures and Tables

**Table 1:** Demographic and clinical characteristics of the study population.

Age, years	52.6 ± 10.7
BMI, kg/m^2^	25.8 ± 4.8
Hypertension, %	32
T2DM, %	4
Dyslipidemia, %	28
Family history of CAD, %	17.2
Active smoking, %	20
Family history of malignancy, %	13.8
Menopause	64

BMI: body mass index, T2DM: type 2 diabetes mellitus, CAD: coronary artery disease. Continuous variables are presented as mean ± standard deviation. Categorical variables are presented as percentages.

**Table 2:** Study population characteristics according to the occurrence of LV dysfunction.

Parameter	LV Dysfunction (*n* = 15)	No LV Dysfunction (*n* = 13)	*p*-Value
Age, years	53.7 ± 11.0	54.5 ± 11.8	0.85
BMI, kg/m^2^	25.0 ± 4.3	27.3 ± 5.1	0.20
Hs Troponin I (ng/L)	2.6 (1.0, 3.4)	1.8 (0.9, 3.0)	0.56
NTproBNP (pg/mL)	84 (64, 118)	73 (54, 147)	0.47
Hypertension, %	26.7	38.5	0.51
T2DM, %	0	7.7	0.27
Dyslipidemia, %	20.0	30.8	0.51
Family history of CAD, %	20.0	15.4	0.75
Active smoking, %	13.3	23.1	0.30
Family history of malignancy, %	20.0	0	0.09
Menopause	66.7	69.2	0.89
HER2 (+), %	86.7	69.2	0.26
Radiotherapy, %	80.0	76.9	0.84
Trastuzumab, %	86.7	69.2	0.26

BMI: body mass index, T2DM: type 2 diabetes mellitus, CAD: coronary artery disease; Hs: high sensitivity; NTproBNP: N-terminal pro B-type natriuretic peptide. Continuous variables are presented as mean ± standard deviation or as median with interquartile range as appropriate. Categorical variables are presented as percentages.

## Data Availability

The datasets supporting the findings of this research are available from the corresponding author upon reasonable request.

## Abbreviations

The following abbreviations are used in this manuscript:

ASE	American Society of Echocardiography
BMI	Body mass index
CAD	Coronary artery disease
CD40	Cluster of differentiation 40
CD40L	Cluster of differentiation 40 ligand
CTRCD	Cancer therapy-related cardiac dysfunction
EACVI	European Association of Cardiovascular Imaging
ELISA	Enzyme-linked immunosorbent assay
GLS	Global longitudinal strain
HER2	Human epidermal growth factor receptor 2
LV	Left ventricular
LVEF	Left ventricular ejection fraction
NF-κB	Nuclear factor kappa-light-chain-enhancer of activated B cells
SPSS	Statistical Package for the Social Sciences
T2DM	Type 2 diabetes mellitus
